# Physical Mapping Integrated with Syntenic Analysis to Characterize the Gene Space of the Long Arm of Wheat Chromosome 1A

**DOI:** 10.1371/journal.pone.0059542

**Published:** 2013-04-16

**Authors:** Stuart J. Lucas, Bala Anı Akpınar, Melda Kantar, Zohar Weinstein, Fatma Aydınoğlu, Jan Šafář, Hana Šimková, Zeev Frenkel, Abraham Korol, Federica Magni, Federica Cattonaro, Sonia Vautrin, Arnaud Bellec, Hélène Bergès, Jaroslav Doležel, Hikmet Budak

**Affiliations:** 1 Faculty of Engineering and Natural Sciences, Sabanci University, Tuzla, Istanbul, Turkey; 2 Centre of the Region Haná for Biotechnological and Agricultural Research, Institute of Experimental Botany, Olomouc, Czech Republic; 3 University of Haifa, Institute of Evolution, Haifa, Israel; 4 Istituto di Genomica Applicata (Institute of Applied Genomics), Udine, Italy; 5 Institut national de la recherche agronomique–Centre National de Ressources Génomiques Végétales (National Plant Genomic Resources Centre), Castanet-Tolosan, France; 6 Sabanci University Nanotechnology Research and Application Centre, Sabanci University, Tuzla, Istanbul, Turkey; Universidad Miguel Hernández de Elche, Spain

## Abstract

**Background:**

Bread wheat (*Triticum aestivum* L.) is one of the most important crops worldwide and its production faces pressing challenges, the solution of which demands genome information. However, the large, highly repetitive hexaploid wheat genome has been considered intractable to standard sequencing approaches. Therefore the International Wheat Genome Sequencing Consortium (IWGSC) proposes to map and sequence the genome on a chromosome-by-chromosome basis.

**Methodology/Principal Findings:**

We have constructed a physical map of the long arm of bread wheat chromosome 1A using chromosome-specific BAC libraries by High Information Content Fingerprinting (HICF). Two alternative methods (FPC and LTC) were used to assemble the fingerprints into a high-resolution physical map of the chromosome arm. A total of 365 molecular markers were added to the map, in addition to 1122 putative unique transcripts that were identified by microarray hybridization. The final map consists of 1180 FPC-based or 583 LTC-based contigs.

**Conclusions/Significance:**

The physical map presented here marks an important step forward in mapping of hexaploid bread wheat. The map is orders of magnitude more detailed than previously available maps of this chromosome, and the assignment of over a thousand putative expressed gene sequences to specific map locations will greatly assist future functional studies. This map will be an essential tool for future sequencing of and positional cloning within chromosome 1A.

## Introduction

It is a truth universally acknowledged that a crop as globally important as bread wheat (*Triticum aestivum* L.) must be one of the highest priorities for genome analysis, to facilitate development of cultivars able to meet the challenges of increasing world population and changing climate. However, its large (16.9 GB) and complex genome has been seen as intractable to whole genome sequencing. *T. aestivum* is a hexaploid species (6n = 6× = 42) believed to have originated from two consecutive hybridization events involving three diploid wheat ancestor species; first between *T. monococcum* and a second wild diploid wheat (possibly *T. searsii*) to produce the AABB allotetraploid *T. turgidum*; then more recently with diploid *Aegilops tauschii* to give the AABBDD allohexaploid genome. Therefore the *T. aestivum* genome is both triple the size of related diploid species such as barley, and complicated by having three similar but divergent paralogs of many genes, one descended from each ancestral species. A further confounding factor is that, as with other very large grass genomes, its majority is comprised of repetitive elements (85–90%) with only a little over 1% encoding transcribed genes [1]. Bearing these factors in mind, even though new generation sequencing technologies make it feasible to obtain reasonable coverage of such a large genome, assembling the resulting short sequence reads seems an insoluble problem.

In view of this, the International Wheat Genome Sequencing Consortium (IWGSC; www.wheatgenome.org) has proposed a chromosome-by-chromosome approach to mapping and sequencing bread wheat, enabled by recent improvements in chromosome flow-sorting technology. One pre-requisite for generating a reference sequence of each chromosome is a high-density physical map to act as a scaffold for sequence assembly. In addition to their utility for genome sequencing, physical maps are a valuable resource for map-based cloning of genetic loci encoding agriculturally important traits. Therefore, locating regions of the chromosome that encode expressed genes is of particular importance. To date, the available physical map of the long arm of wheat chromosome 1A is limited to just three deletion bins [2].

Physical mapping using bacterial artificial chromosome (BAC) clone libraries can generate maps of a genome which lacks extensive polymorphism and facilitate studying its structure. The previously published physical map of wheat chromosome 3B [3] demonstrates the effectiveness of this approach for *T. aestivum*. BAC libraries are constructed from DNA of single chromosomes or single chromosome arms, isolated by chromosome or chromosome arm flow sorting [4]. Restriction fragment sizes are then used to generate a unique fingerprint for each BAC clone, which can be assembled into physical contigs. Diverse molecular markers are then used to anchor the contigs to genetic maps and establish their order along the chromosome.

The availability of genome sequences of rice, sorghum and the model grass *Brachypodium distachyon* has revealed that a substantial group of genes are very well conserved across grass genomes. Therefore, gene colinearity between the Triticeae and the model grasses for which a reference sequence is available, can be an effective and accessible method for dissecting the larger and more complex chromosomes, as shown by the construction of a virtual gene order for chromosomes of barley [5].

In order to use these resources in a co-ordinated way to improve knowledge of the wheat genome, the EU-FP7 TriticeaeGenome project (www.triticeaegenome.eu) was established in 2008 with 17 international partners. One of the major goals of the project was to produce anchored physical maps for all the homoeologous group 1 and group 3 chromosomes of bread wheat and barley, in addition to refining the map of chromosome 3B that had already been produced [3]. This project has promoted the development of new mapping resources such as a new tool for physical contig assembly, Linear Topology Contig (LTC, [6]) and new types of molecular markers designed for large, repetitive genomes [7], [8]. New high-throughput methods have been developed to facilitate genetic anchoring of BAC-based physical maps for large plant genomes (reviewed in [9]). While most of the physical maps generated by the project have not yet been published, they are already being used to advance a number of gene and QTL mapping projects [10]. The wheat database established at INRA URGI (http://urgi.versailles.inra.fr/Species/Wheat) enables users to explore the group 1 & 3 chromosome physical maps in relation to other resources, such as 26 wheat genetic maps, using a Gbrowse interface [10].

Under the umbrella of this project, we present the physical map for the 523 MB long arm of *T. aestivum* chromosome 1A (1AL) using fingerprint data from 70,537 BAC clones. Chromosome arm 1AL is known to harbour the *Glu-A1* locus which is important in determining the end-use quality of bread wheat [11], as well as other genes involved in metabolism and disease resistance [12]; however, only the first of these genes has been cloned. A Minimum Tiling Path (MTP) of 7470 clones was generated, and BAC-end sequences (BES) obtained for the MTP clones (see [1]). From the MTP clones, a PCR-based 3D pooling approach was used to identify clones containing 365 molecular markers for anchoring the contigs to genetic maps. Furthermore, hybridization of the BAC pools to a wheat transcriptome microarray was used to identify 1122 expressed genes encoded by chromosome 1AL, and locate them on the physical map. In addition to integrating the physical map with existing genetic maps of this chromosome, the co-linearity of conserved grass genes was exploited to produce a syntenic map of 1AL by comparison to *B. distachyon,* Sorghum and rice. The resulting map provides the most comprehensive study to date of genetic loci on this chromosome, and will be an essential tool for positional cloning within it and future assembly of a reference sequence.

## Results

### Construction of BAC-based physical maps of chromosome 1AL

**Figure 1:**
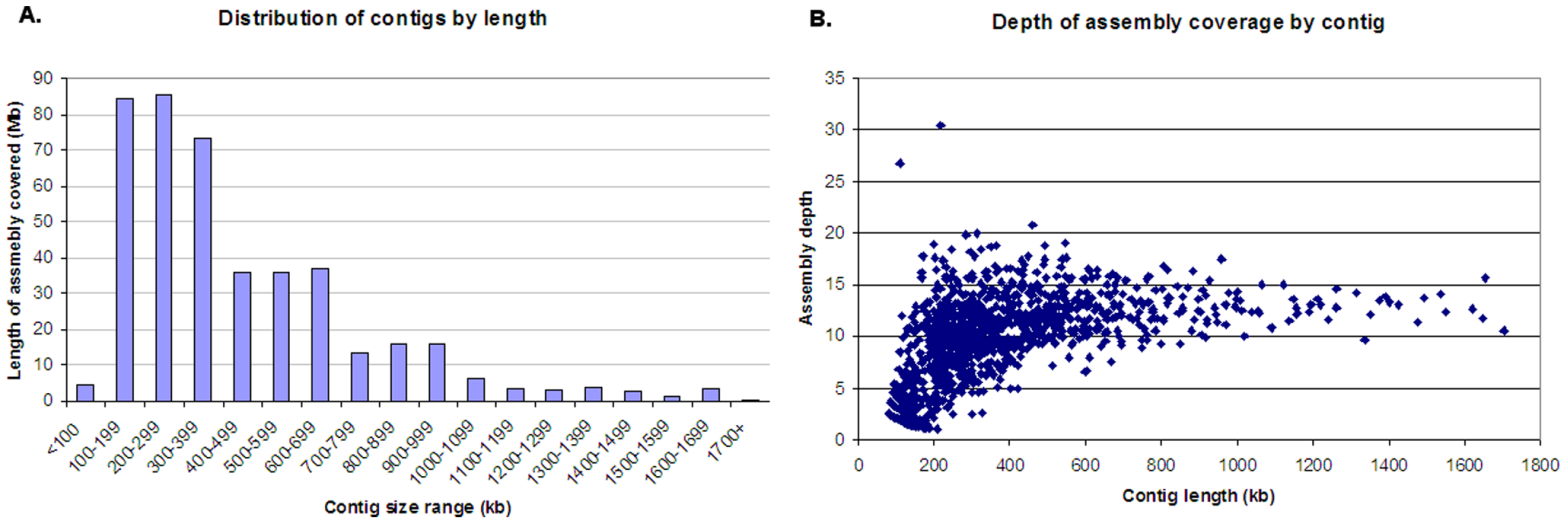
Contig length and depth distributions in the 1AL physical map. **A.** Contigs were grouped by estimated length into blocks of 100 kb, and the total length of each block calculated and plotted. The length of each contig was estimated from the fingerprints using an average consensus band length of 1200 bp. **B.** The depth of coverage of each contig was calculated by multiplying the number of clones in the contig by the average BAC insert size (105 kb) and dividing by the contig length.

Our previous study [1] reported the production of BAC libraries covering chromosome 1AL to a depth of >15× coverage, with an average insert size of 105 kb. Good quality fingerprints were obtained for 70,537 (76.2%) of these clones by High-Information Content Fingerprinting (HICF), and then assembled using FingerPrinted Contig (FPC) software (see materials and methods). After the completion of a primary automated assembly, the build consisted of 2551 contigs with an average size of 233 kb and a total length of 623 MB (not including the 20,807 singletons). The Minimum Tiling Path (MTP) was generated from this assembly and used for marker development and anchoring (see below). The build was then edited manually to merge similar contigs at lower stringency and incorporate information gained from molecular markers, reducing the number of contigs to 2210. Unanchored small contigs are deemed to be uninformative, so these were removed to give a final assembly of 1180 contigs (Table 1) and a total length of 446 MB (85.3 % of the predicted size of 1AL). The quality of the assembly was assessed by plotting the size distribution and depth of coverage of the contigs (Figure 1). As shown in Figure 1A & Table 2, the largest contig was 1.78 MB in size; there were 36 contigs of greater than 1 MB, with increasing numbers of contigs at smaller size ranges. The N50 value (the smallest contig size required to cover 50% of the assembly) was 460 kb; L50 (starting with the largest, the number of contigs required to cover 50% of the assembly)  = 310 contigs. The average depth of coverage across the whole assembly was 10.9×, and the depth of individual contigs clustered around this value, with larger contigs tending to have slightly higher coverage (Figure 1B). Five short (<N50) contigs had a coverage of 20× or greater, suggesting that they may consist of large duplications that have been condensed into one contig.

**Table 1:** Summary of assembly process of 1AL BAC clone fingerprints.

Physical map construction		No. of contigs	No. of singletons	Assembly length (kb)^1^	N50 (kb)^1^
Automated assembly	Initial build of all fingerprints (cutoff 1e-75)	5737	30990	865109	-
	Add singletons & merge (cutoff 1e-70)	4991	28801	801774	-
	Add singletons & merge (cutoff 1e-65)	4264	26657	742893	-
	Add singletons & merge (cutoff 1e-60)	3662	24824	695190	-
	Add singletons & merge (cutoff 1e-55)	3272	24413	664550	-
	Add singletons & merge (cutoff 1e-50)	2810	22068	636733	-
	Final build before manual editing (cutoff 1e-45)	2551	20807	622795	277
Manual marker and small overlap-based merging of contigs (cutoff 1e-25)		2210	20867	596581	341
After removal of small contigs^2^		1180	26193	446042	460

1 Calculated based on an average consensus band length of 1.2 kb; does not include singletons.

2 Defined as comprising <6 BAC clones and/or <200 kb in length, with no markers. Clones from these contigs are added to the list of singletons at this stage.

**Table 2:** Comparison of assembly statistics by FPC and LTC methods.

Assembly algorithm	FPC	LTC
Number of contigs (>5 clones)	1180	583
Total number of clones in contigs	44344	49882
Number of singletons	26193	20655
Estimated chromosome coverage^1^	85%	88%
Largest contig^1^	1.78 Mb	6.05 Mb
N50 contig size^1^	460 kb	1166 kb
L50 contig number	310	133

1 Calculated based on an average consensus band length of 1.2 kb.

Accurately assembling BAC clone fingerprints is complicated by some clones giving low quality fingerprints, and the presence of chimeric BAC clones that contain segments from two different parts of the chromosome. Moreover, the 1AL BAC libraries used in this study are estimated to be 15% comprised of contaminating sequences from other chromosomes – these probably account for a significant number of the >20,000 singleton BACs that could not be incorporated into the assembly. Linear Topology Contig (LTC) software is a recently developed tool that exploits the fact that physical chromosomes are linear; therefore, accurately assembled contigs should also be linear in structure, rather than branched or circular. LTC has been shown to give superior results to FPC in assembling simulated datasets derived from rice and maize [6]. Therefore, in addition to the FPC assembly, LTC was also used to generate a second version of the physical map. Five hundred and seventy-six contigs were generated in the first round of adaptive clustering, after the removal of 1490 Q-clones (questionable clones) from the analysis. Eleven of these contigs were highlighted as non-linear by LTC, which led to a further exclusion of 46 clones from the analysis to eliminate discrepancies in contig topology. After removing these clones, a second and final round of adaptive clustering by LTC yielded 583 contigs, each containing 6 to 669 clones. In the final LTC assembly, 3 contigs – namely cl_20433, cl_20634 and cl_20677– were still indicated to have non-linear topology, which most likely resulted from the inclusion of one or two relatively low quality fingerprints in the MTP (Figure S1).

A brief comparison of the FPC and LTC assemblies of 1AL is given in Table 2. While the LTC assembly gave a similar estimated coverage of the chromosome arm to FPC (88% as opposed to 85%) it clearly generated much longer contigs. The number of contigs in the final assembly and the minimum number required to cover half of the assembly (L50) were both less than half that of FPC, while the N50 contig size was more than twice as large. Furthermore, LTC was able to incorporate >5000 more BAC fingerprints into contigs of at least 6 clones than FPC. These relatively larger contig sizes make the LTC assembly attractive; however, experimental verification is required to determine whether this assembly is more accurate than that produced by FPC. Furthermore, the 2 programs use different methods for selecting MTP clones and so give very different results. As the MTP from FPC had been determined and used for contig anchoring (below) before LTC became available, the rest of this study refers to the FPC assembly; however, future studies may wish to compare the two assemblies when exploring a particular region of the chromosome Both physical maps, including the unanchored small contigs, are available for browsing at http://urgi.versailles.inra.fr/Species/Wheat.

### Anchoring of contigs by molecular markers

**Figure 2:**
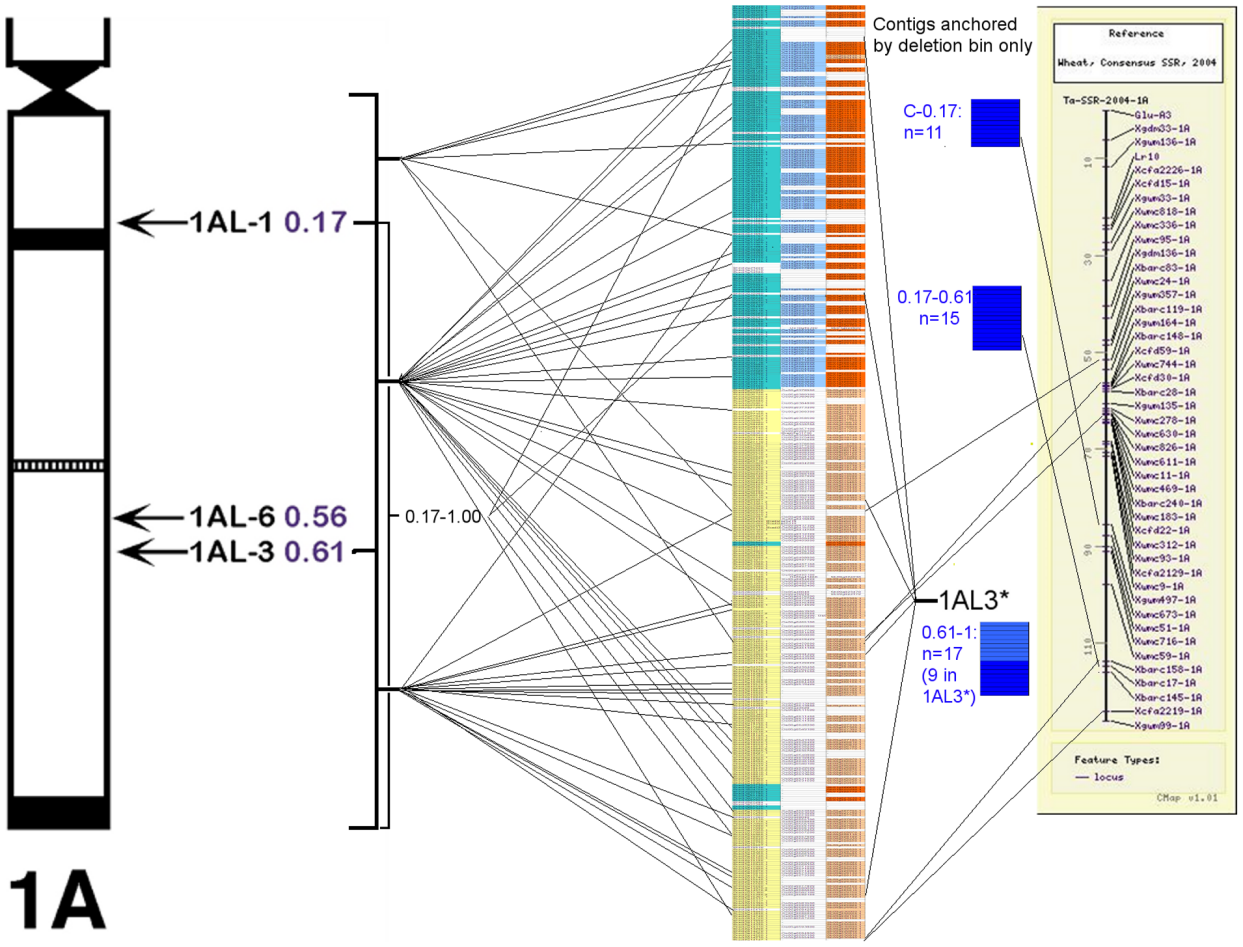
Comparison of the synteny-based contig order with deletion bin and SSR maps. The contig order of the synteny-based physical map (centre) is related to the established deletion bins for chromosome 1AL (left) and the consensus SSR map for chromosome 1A by Somers et al (right, [13]). Each horizontal line on the synteny-based map represents a single contig, while the connecting lines show deletion bin assignments/genetic markers found on the same contig. Contigs that were located in deletion bins but not in the synteny-based contig order are shown immediately to the right of the synteny-based map. For color marking of the synteny-based contig order, see Fig. 4.

The BAC clones comprising the MTP were re-gridded into a sub-library on 384-well plates, which was then PCR screened for molecular markers using a 3D pooling approach. From previously published 1AL genetic maps, 38 SSR markers and 21 EST markers were tested, however only a minority of these (12 SSRs and 10 ESTs) could be successfully assigned to specific clones. One reason for the comparatively low success rate of assigning these markers could be sequence differences between different wheat lines. Therefore, further anchoring was carried out using Conserved Orthologous Set (COS) markers, designed by identifying conserved genes in the sequenced grass species that show high sequence similarity to wheat ESTs previously mapped to chromosome 1AL [7]. In addition to the 48 1AL COS markers already published, a further 350 COS markers were screened that had been designed by comparison of wheat chromosome 1AL survey sequences to *Brachypodium,* sorghum and rice coding regions (J. Salse et al, 2013, manuscript under review). After screening the 3D pools, 156 COS markers were assigned to specific BAC clones. These markers are particularly useful as they anchor regions of the physical map that encode genes; however, a large proportion of chromosome 1AL is made up of transposable elements, especially long terminal repeats. To anchor parts of the physical map that might lack genes, BAC-end sequences (BES) were obtained for the MTP clones and used to develop insertion site-based polymorphism (ISBP) markers [1]. These markers are designed to take advantage of the unique sequence junctions found where transposable elements have integrated into the chromosome [8]. In addition to the 23 ISBP and SSR markers reported previously [1], a further 280 ISBP markers were selected from the BES, of which 164 were successful in screening of the 3D pools. The ‘unsuccessful’ markers either failed to amplify in PCR reactions, or were non-specific in BAC pool screens. In contrast, most of the 164 specific markers were useful in confirming overlaps between the clone from which they had been designed and the adjacent MTP clone, or identifying overlaps that had not been detected in the fingerprints. Details of the primers and map locations for all the new markers are given in Table S1. All of the novel SSR and ISBP markers were also screened against chromosome 1AL deletion lines, and 67 (35.8%) were found in specific deletion bins. Interestingly, a subset of the markers mapped to deletion bin 1AL3 (0.61–1.00) were also present in deletion line 4511–18 (1AL6), which according to cytogenetic analysis is shorter than 1AL3, being truncated to 0.56 of the full chromosome length. All markers mapped to the centromeric region or deletion 1AL1 (0.17–0.61) were also present in this line. Therefore we suggest that part of the truncated 1AL sequence has in fact been translocated to a different chromosome (there is also a deletion in 2BS in this line). In any case, markers deleted in 1AL6 appear to comprise up a subset of those in 1AL3, which was designated 1AL3*. This data was combined with previously bin-mapped EST and SSR markers, and used to construct a deletion bin map of 112 contigs (Figure 2) which was compared with the syntenically-derived contig order (below) and the consensus SSR map for this chromosome [13]. While the deletion map and syntenic map were broadly consistent, there were differences, and there was some overlap between the deletion bins on the syntenic map. In particular, a section of the syntenic order (103–115 cM from the centromere) contained five markers for deletion bin 1AL1 and none for 1AL3, to which the regions flanking it could be tentatively assigned; this may indicate a chromosomal translocation, although a greater number of deletion bin markers would be required to determine this. Of the 14 contigs assigned to bin 1AL3*, only 5 contained syntenic genes and these were widely dispersed in the syntenic order, so it was not possible to identify the genetic location of this bin.

### Transcriptome microarray hybridization and syntenic map construction

**Figure 3:**
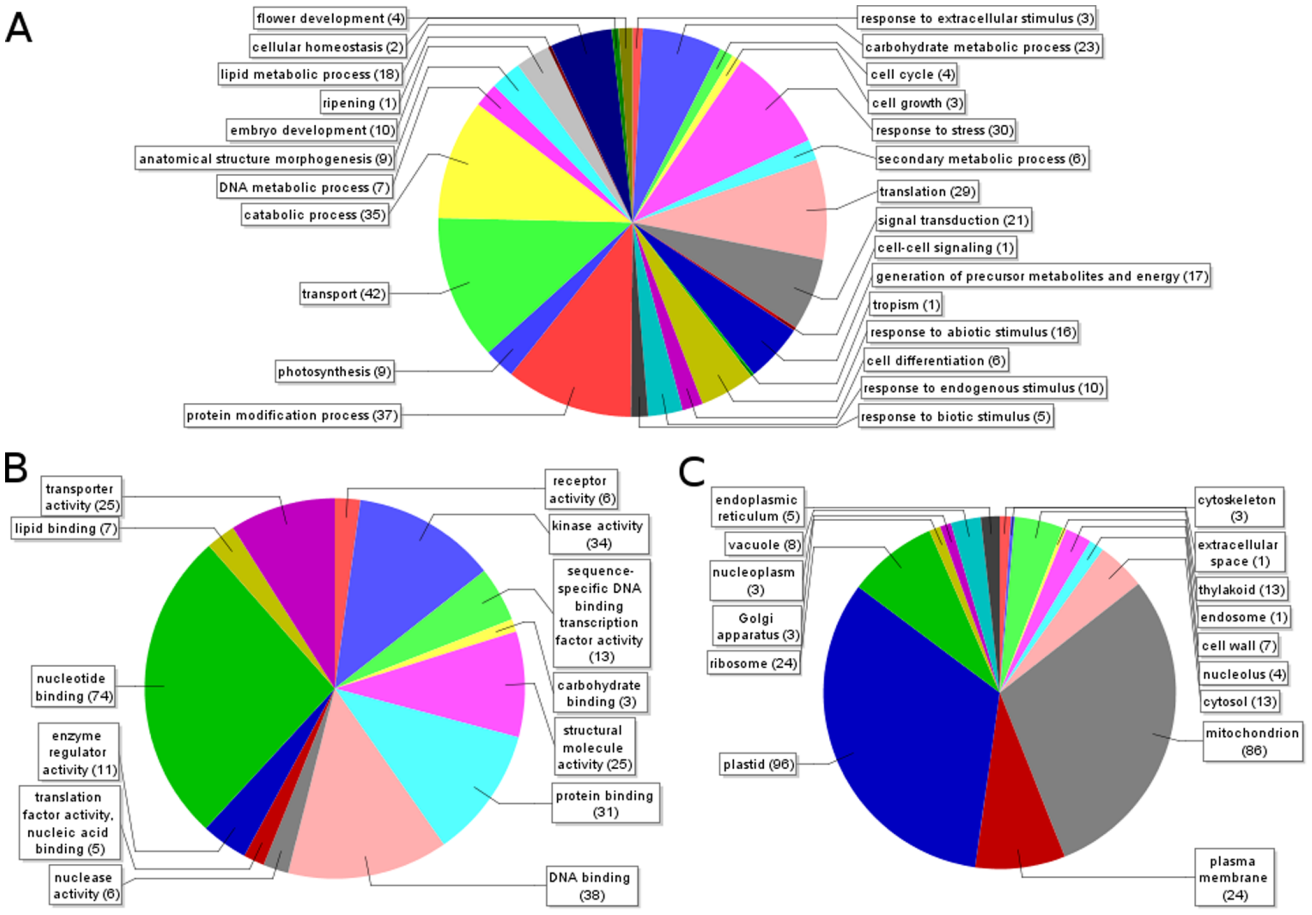
Functional annotation of gene sequences found in 1AL. GO terms were mapped onto the Unigene sequences from the wheat transcriptome 40 k microarray detected in 1AL BACs as described in the Methods section. Multi-level pie charts were constructed showing the most specific GO term assigned for each sequence in three categories: **A.** Biological processes. **B.** Molecular function. **C.** Cellular component. Numbers in brackets show the number of different sequences annotated with each term.

Recently, hybridization of BAC MTP pools to wheat EST microarrays has been shown to be effective for assigning individual genes to wheat physical maps [14],[15]. We used the same strategy to locate genes on chromosome 1AL, by hybridizing the BAC pools individually to a custom NimbleGen microarray comprised of probes for 39,137 wheat Unigenes. After data analysis and deconvolution, 1122 Unigenes were assigned unambiguously to specific BAC clones (Table S2), giving hits on 608 contigs. As these figures suggest, the majority of the Unigenes (776/1122) were found on the same contig as at least one other Unigene. Furthermore, 69% of all the gene-based markers were found on the same or an overlapping BAC clone with at least one other gene marker, consistent with the observation that wheat genes are generally found in gene islands. Taking into account the Unigene assignments with the other markers described above, in total 772 contigs were anchored by at least one molecular marker, and the anchored contigs had a cumulative length of 287.4 MB (67.3% of the whole assembly). The mapped Unigene sequences were then functionally annotated using Blast2GO (see Materials and Methods). Out of 1122 Unigenes, 735 had at least one BLAST hit to a known protein and, following automated GO mapping, 459 of these were annotated with one or more GO terms; all annotations were added to Table S2. The distribution of GO annotations is summarized in Figure 3; Biological Process terms were assigned to 349 sequences, with the most prevalent being transport (42 sequences), protein modification (37 sequences) and catabolic processes (35 sequences). Molecular Function annotations were made for 278 sequences, with nucleotide binding the largest group (74 sequences). A further 56 sequences were associated with DNA binding, transcription or translation factor activity, indicating that gene regulation makes up a major component of the functions of the genes detected on chromosome 1AL. A total of 291 sequences had Cellular Component terms, including a surprising predominance of mitochondrial (86) and plastid (96) proteins. This may reflect the fact that the complete plastid and mitochondrial genomes of both wheat and barley have already been sequenced, and therefore more complete annotations are available for mitochondrial and plastid genes that may have been duplicated and transferred to the nuclear genome.

Another approach that has proved highly effective for analysing large grass genomes is low-pass survey sequencing combined with syntenic analysis. Chromosome 1AL has recently been sequenced at 1.5× coverage using 454 technology [16], and similarity searches using these sequences identified 1352 genes that are conserved and mostly co-linear with the genomes of *Brachypodium,* rice and sorghum. These genes have been used to construct a ‘genome zipper’ of chromosome 1AL giving a virtual order of syntenic sequences [5], based on the gene order in *Brachypodium,* the most closely related of the three species. Syntenic sequences that also match genetically mapped markers, enable genetic distances to be incorporated into the genome zipper.

**Figure 4:**
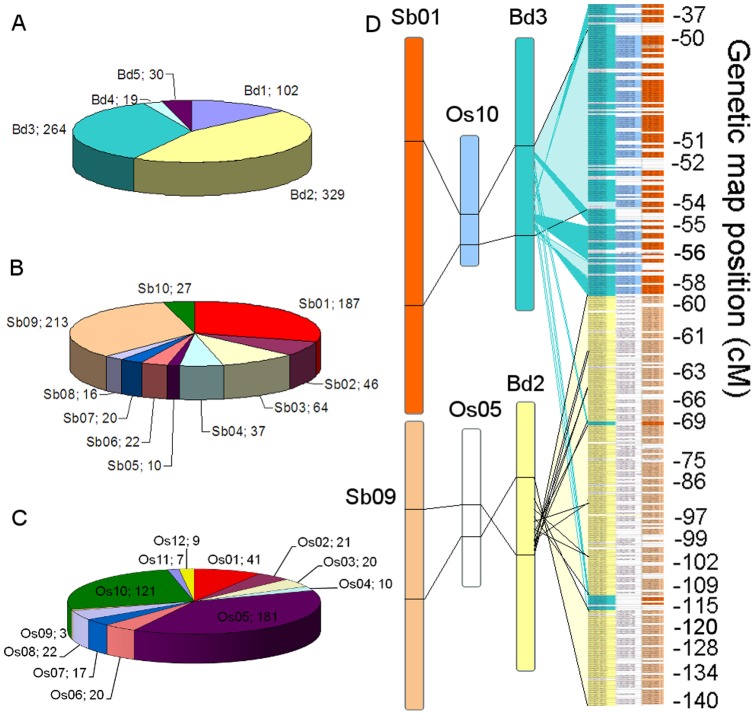
Relationship between gene-based markers on 1AL and conserved genes in sequenced grass species. **A.** Chart showing the number of gene-based markers found to have homologs on each *B. distachyon* chromosome by similarity searches. **B.** Chart showing the number of gene-based markers found to have homologs on each *Sorghum bicolor* chromosome by similarity searches. **C.** Chart showing the number of gene-based markers found to have homologs on each *Oryza sativa* chromosome by similarity searches. **D.** The contig order of the synteny-based physical map is shown (right), with genetically mapped marker positions marked in cM. Each horizontal line represents a single contig on the physical map. Connection lines show the locations of syntenic regions on *B. distachyon* chromosomes 3 & 2, and equivalent regions on *Oryza sativa* chromosomes 10 & 5, and *S. bicolor* chromosomes 1 & 9.

The sequences of all the Unigenes that had been assigned to contigs, along with the EST & COS markers present on the physical map and conserved gene sequences previously identified in the BES [1] were compared with the genes from sequenced grass species by sequence similarity searches (Figure 4). Out of a total of 1337 gene based markers, 745 were homologous to *B. distachyon* coding sequences (Figure 4A) while 643 had homologs in *Sorghum bicolor* (Figure 4B) and 472 had homologs in *Oryza sativa* (Figure 4C). In each case the largest number of hits were to the chromosomes known to be syntenic to the long arm of wheat chromosome 1– *B. distachyon* chromosomes 2 & 3, *S. bicolor* chromosomes 1 & 9, and *O. sativa* chromosomes 5 & 10. For *B. distachyon,* the co-linearity with 1AL is observed in the regions encompassing genes Bradi2g13750– Bradi2g29960 on chromosome 2, and Bradi3g19747– Bradi3g34260 on chromosome 3. However, matches were also found on these chromosomes outside these co-linear regions; moreover there were also hits to all the other chromosomes, suggesting while a large number of 1AL genes are co-linear with other grass genomes, many non-syntenic genes or gene fragments are also present. All markers which corresponded to a gene from the syntenic regions of at least one of the 3 sequenced grass genomes (432/1337) were compared with the 1AL genome zipper; syntenic genes were identified on the physical map, enabling 310 contigs to be placed in syntenic order (Fig. 4D). In 60 cases, two different markers gave matches to the same syntenic gene; 43 of these marker pairs had also been independently assigned to the same BAC clone, giving confidence in the consistency of the different methods used to identify gene-based markers. From the remainder, 8/17 pairs were assigned to the end clones of 2 different contigs. These were presumed to correspond to hits to different parts of the same gene on 2 BAC clones with too short an overlap region to have been assembled (approximately 25 kb overlap is required), and thus the pairs of contigs were merged. The remaining 9 pairs may represent gene duplication events. Similarly, 91 genes with near neighbours also present in the syntenic map were found on the same contig as those neighbours, demonstrating that the *Brachypodium* syntenic gene order is broadly conserved in wheat and useful for structuring the physical map.

### Verification and refinement of the 1AL physical map by contig topology

**Figure 5:**
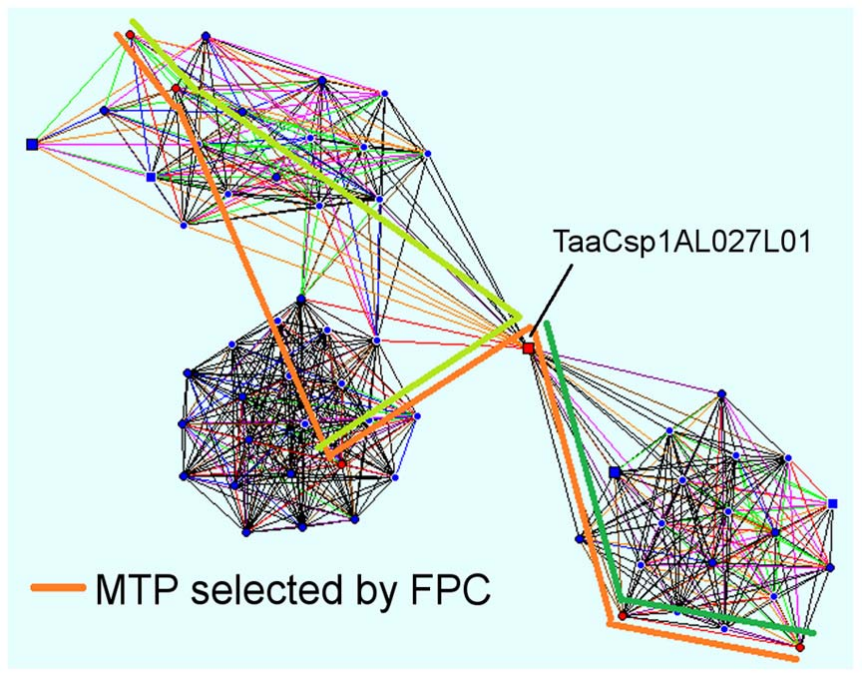
Using Linear Topology to resolve problematic contigs. Contig205 from the 1AL physical map is represented as a net of significant clone overlaps using LTC software [6], with each vertex representing a clone, and each line a significant overlap between 2 clones. Red circles represent MTP clones, while squares denote clones that have no direct overlap with the MTP. Shapes with a white border denote buried clones. Indicated clone TaaCsp1AL027L01 has multiple significant overlaps with clones in both the left and right halves of the contig, and so has been selected as an MTP clone by FPC (orange bar). However, the fact that no other clones connect the 2 halves of the contig suggest that this may be a chimeric clone, and the contig should be split. Possible MTP routes for the 2 resulting contigs are indicated by the light and dark green bars.

The LTC program described above also provides functionality for testing the linearity and integrity of contigs generated by FPC. This was used to evaluate the physical map of 1AL. First of all, the MTP generated by FPC was checked for inconsistencies by assessing the 2551 contigs produced at the end of the automated assembly process (Table 1). LTC was used to search these contigs both for non-significant clone overlaps, and for incorrectly ordered clones. By this approach, 763/5071 (15%) of MTP clone overlaps selected by FPC were found not to be significant at the recommended cutoff of 10^−15^ [6]. This does not usually indicate problems in the MTP, but reflects the different methods used to select MTP clones by the 2 programs: LTC requires a significant overlap between each pair of MTP clones at the specified cutoff, whereas the procedure used by FPC incorporates information from spanning and flanking clones to confirm the MTP selection [17], allowing relatively shorter overlaps at the conditions selected (see Materials and Methods) and therefore fewer clones to be selected to cover the whole assembly. Accordingly, for 536 out of 548 contigs reported to contain non-significant MTP clone overlaps, LTC confirmed the MTP clone order calculated by FPC. This high degree of consistency between the 2 algorithms gives confidence in the accuracy of the assembly. For the remaining 12 contigs, plus 2 more that passed the clone overlap test, inconsistencies were detected in the ordering of the MTP clones; this could be due to redundant overlaps between MTP clones (in which case the order cannot be determined) or non-linear topology of the respective contig. For example, the sequence of MTP clones in contig205 was different when re-analyzed by LTC compared to that given by FPC; also, the 2 clones in the middle of the MTP whose order was reversed (202E18 and 027L01) had small overlaps both with each other and their flanking MTP clones. Examining the net of significant clone overlaps generated by LTC for this contig (Figure 5) indicated that the contig is branched at clone 027L01, suggesting that this is likely to be a chimeric clone. If this clone is excluded and the contig reassembled by FPC, it splits into two separate contigs, and a new MTP can be selected to cover each. This illustrates the way that inconsistency between the FPC and LTC assemblies can be used to highlight potential problematic clones, and avoid spending resources on them during mapping and sequencing projects. A complete list of the results of verification of MTP clones is given in Data S1.

The final, manually edited assembly contained 2210 contigs before the removal of short contigs (<6 clones) and these were also evaluated using LTC. Overall the great majority of contigs (2156/2210) were verified to be linear in structure. The 54 that were not verified had a total length of 42.8 Mb (7.2% of the assembly); of these, 39 were reported to contain a break in the middle of the contig (at cutoff 10^−15^ of Sulston score). All of these cases were also indicated to contain a break by FPC at this cutoff, but 27 had been joined manually by the presence of the same marker in two non-overlapping clones. These represent pairs of clones that do overlap physically, but with too short an overlap to have significantly similar fingerprints. The remaining 15 contigs (12.5 Mb, 2.1% of the assembly) were highlighted as being non-linear in structure. Examination of the net of significant clone overlaps for these contigs enabled problems to be identified, such as putative low-quality fingerprint clones, chimeric clones and incorrect MTP selections. In several cases, the apparent non-linearity could be resolved simply by adding a clone to the MTP (see Data S1 for a list of these contigs, and Figure S2 for examples of each kind of non-linearity).

Apart from problems at the level of contig structure, both FPC and LTC identify clones with questionable fingerprints, labelling these as Q-clones. FPC defines Q-clones as those which fit poorly with the contig into which they are assembled; a contig containing more than 10% Q-clones is considered to be mis-assembled, and all such contigs are eliminated at each stage of the automated assembly with FPC. However, contigs with fewer than 10% Q-clones remain, such that in the final FPC assembly a total of 1709 Q-clones (3.8% of assembled clones) remain, distributed between 616 contigs. In contrast, LTC defines Q-clones as those with apparently significant overlaps with more than one otherwise non-overlapping cluster of clones; therefore Q-clones are putatively chimerical, and are excluded during contig assembly. When evaluating the FPC contigs, LTC detected 1229 Q-clones (2.8%) distributed among 665 contigs. They were more prevalent in longer contigs, such that the total length of contigs containing one or more Q clones was 313.8 Mb, or over half of the assembly. Future fine-mapping studies of these contigs should consider avoiding the Q-clones (listed in Data S1); however, as some may show true overlaps in parts of the chromosome with low coverage in the BAC libraries, they have not been removed from the assembly.

Overall, however, validation of the large majority of FPC contigs and clones by LTC is encouraging, suggesting that these assemblies correctly reflect the physical state of the chromosome.

### Characterization of the gene space of chromosome 1AL

**Figure 6:**
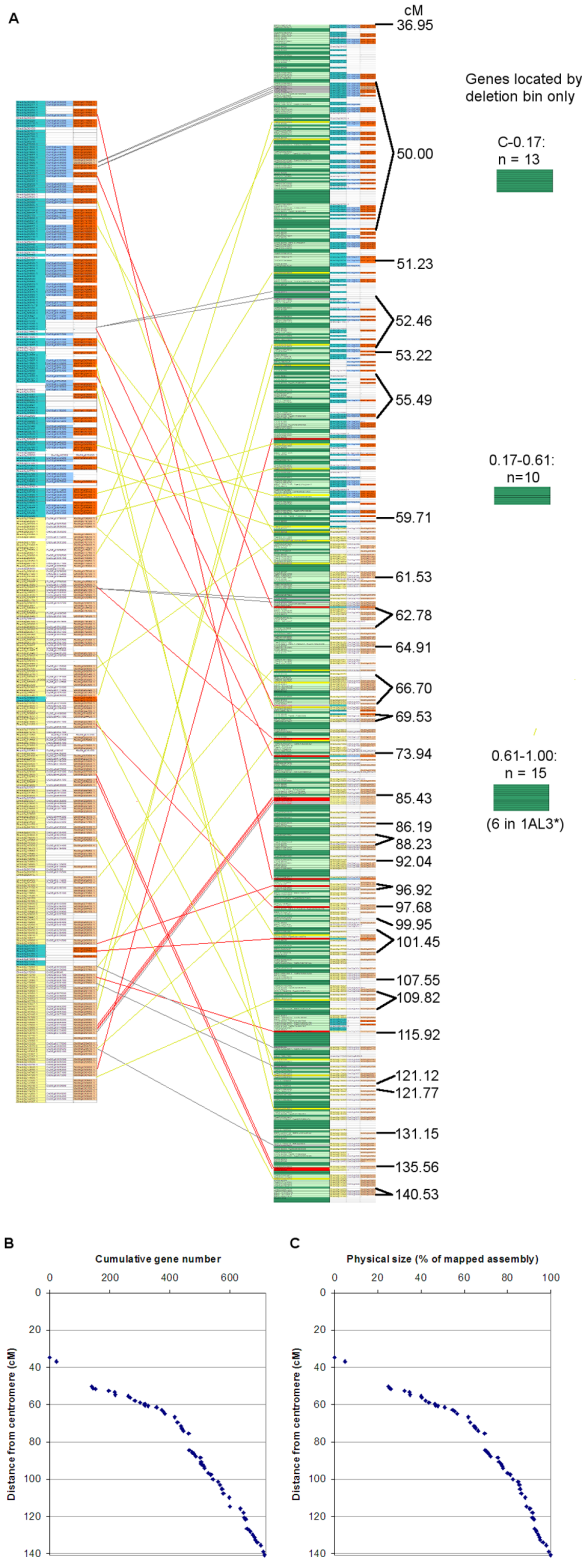
Representation of the physically mapped gene space of chromosome 1AL. **A.** The syntenically predicted gene order (left) is compared with all gene markers found on physically mapped contigs (centre). Each horizontal bar represents a single gene; syntenic genes are shaded in pale green, non-syntenic genes in dark green. Connections show syntenic genes found in unexpected locations on the physical map; grey connections and bars indicate putative gene duplications. Red connections and bars indicate translocations of individual genes to a different genetic location. Where two genes from different locations of the syntenic sequence were found on the same contig, and no other evidence was available to determine which location is correct, the genes are marked and connected in yellow. The genetic ruler indicates gene markers that have defined genetic distance from the centromere, while others were interpolated using the syntenic sequence. **B.** Using the gene sequence shown in **A,** cumulative number of gene markers was plotted against genetic distance from the centromere. **C.** Using the gene sequence shown in **A,** cumulative length of contigs harboring syntenic genes was plotted against genetic distance from the centromere.

The syntemically ordered physical contigs described above were used as a basis for constructing a map of the gene space of chromosome 1AL (Figure 6). Non-syntenic genes located on contigs with syntenic genes were incorporated; 25 contigs harboring genes conserved with *B. distachyon,* which were themselves absent but had flanking genes present in the syntenic gene order, were added into the map. When 2 genes predicted to be widely separated by the syntenic map were found on the same physical contig, these cases were examined in detail. Some were attributable to mis-assembled contigs; 11 contigs were split (and 1 subsequently merged with a different contig) for this reason. After eliminating these instances, 24 genes were found in locations different from those predicted by the syntenic gene order (indicated by red and yellow connection lines in Figure 6A). These are likely to correspond to rearrangements that have taken place in chromosome 1AL since wheat diverged from *B. distachyon.*

In total, 716 genes were located in the syntenically ordered gene space; a further 38 genes were found in deletion bins but could not be assigned a genetic or syntenic location. The non-syntenic genes were distributed throughout the map, interspersed with the syntenic genes (indicated by dark and light green bars respectively on Figure 6A). Putative translocations were dispersed throughout the length of the map, although multiple rearrangements appear to have a occurred at 85.43 cM from the centromere. Two genes expected to be at this location were found close to the telomere, at a genetic map position of 135.56 cM, while 4 genes predicted to be at 128.14 cM were in fact found at 85.43 cM. The most stable region of the chromosome appears to be 50 cM from the centromere, where 111 genes are found that could not be distinguished by genetic distance and only 2 of the putative translocations are observed. The cumulative gene number and physical size of the contigs in the gene space map were plotted against genetic distance (Figure 6B, C). The shallower gradient of the graph near the centromere indicates that there is a higher density of genes relative to the genetic distance in this region. There appears to be a discontinuity with very few genes in it between 75 and 85 cM, the end of which corresponds to the multiply rearranged segment mentioned above. After this point the gene density is lower than before and remains fairly uniform to the telomeric end of the map (Figure 6B). The cumulative length of the mapped physical contigs (Figure 6C) matches the gene number curve extremely closely. This shows that, on a large scale, the frequency of genes per physical distance is fairly uniform across the length of 1AL; therefore the higher gene density relative to genetic distance is a result of less recombination in the centromeric region of the chromosome arm, correlating with their being relatively few relocated genes in this region. Overall, the conservation of gene order between the syntenic genes of chromosome 1AL and sequenced grass genomes is striking.

## Discussion

The construction of high-density physical maps is a major milestone in the international effort to sequence the genome of wheat, the most widely grown of the world's food crops. Until recently, the only physical maps available for wheat were cytogenetic deletion bins, with necessarily limited resolution given the large size of the wheat genome. In 2008, the first BAC library-based physical map of a wheat chromosome 3B was published [3] and projects are now in progress to map each of the remaining wheat chromosomes by the same strategy. This study presents the first BAC library physical map of *T. aestivum* chromosome 1AL, which along with the map of 1AS (Breen *et al*., in preparation) gives a complete picture of this chromosome.

The total length of the final assembly is 446 MB, 85% of the size estimated from the mass of the chromosome arm. This is a very similar proportion to that observed for the map of chromosome 3B (82%). However, the generation of the map from a 15x coverage BAC library means that the actual coverage of the chromosomal sequences should be much closer to 100%, although some regions may be refractory to characterization by BAC fingerprinting. The discrepancy in sizes is most likely due to errors in the different methods of estimating chromosome size, but there is also the possibility that large scale duplications could lead to two similar regions of the chromosome being assembled into a single contig. Analysis of the clone depth of each contig (Figure 1B) identified a few contigs which appear to have >20 fold coverage, which are therefore suspects for such mis-assembly. Focusing on these suspect contigs will enable further refinement of the assembly.

The final FPC assembly of chromosome 1AL was tested with the recently developed LTC software [6] to evaluate and enhance the quality of the physical map. LTC software reported non-significant overlaps between adjacent MTP clones of 548 contigs. This is not usually a concern, because FPC uses additional information from spanning and flanking clones [17] to determine the MTP, allowing smaller overlaps between MTP clones. Accordingly, for 536 of these, the order of clones was verified by LTC, thereby justifying the MTP selection; however, future fine mapping of these contigs might be facilitated by adding bridging clones between pairs of MTP clones if the overlaps cannot be confirmed. The order of MTP clones in 14 contigs re-analyzed by LTC was not consistent with that calculated by FPC. This situation is likely to result from redundant overlaps given by buried or chimeric clones, which render it impossible to determine the clone order precisely, or cause local non-linearity within contigs. Additionally, LTC detected 39 putatively not-connected and 15 non-linear contigs in the final FPC assembly. The majority of not-connected contigs (27/39) had been merged by the presence of shared molecular markers, even though their fingerprints did not overlap. This can be expected to occur if two clones have relatively short physical overlaps (<20 kb) that contain the marker sequence but are too short to produce significantly similar fingerprints. Topologically non-linear contigs, on the other hand, may result from chimeric clones, low quality fingerprints or incorrect merging of the contigs during the primary assembly. In addition, 56 FPC contigs were judged to contain >10% Q-clones (defined as potentially chimeric) by LTC. Closer investigation of these contigs (Data S1) is recommended if mapping of an agronomically important trait located on them is desired. However, overall LTC verified the structure of 92% (1083/1180) of the FPC contigs, giving confidence that the assembly accurately reflects the physical structure of the chromosome. LTC was also used to generate a secondary contig assembly from scratch, and gave a similar coverage of the chromosome arm but generally produced longer contigs, such that the final contig number (583) was less than half that of FPC. These results illustrate the value of comparing the LTC and FPC methods to identify inconsistencies in contigs, and the lower contig number from the secondary assembly suggests that LTC is an effective tool for assembling fingerprints from complex, repetitive genomes. However, sequencing of contigs generated by each of the two methods is required to confirm which is the most accurate; in this study, the FPC-derived assembly had been used as the basis of the physical map, following guidelines established by the TriticeaeGenome project.

The physical map was anchored using 365 molecular markers of a variety of types (SSR, EST, COS and ISBP) as well as by assigning 1122 expressed genes to chromosome 1AL by hybridization to a wheat transcriptome microarray. Even with such a large number of markers, only 302/1180 contigs present in the final assembly were anchored to genetic or syntenic maps; however, more than two-thirds of the assembly length was covered by at least one molecular marker. The majority of the markers used in this study were based on gene sequences, so the unanchored contigs are likely to contain gene-poor regions of the chromosome, as only the 199 SSR and ISBP markers represented anchor points for non-coding DNA. A large number of additional non-coding markers will be required to anchor the rest of the contigs. However, given the observation that wheat genes are usually found in islands [18], it is likely that the majority of 1AL genes are located on the contigs which have already been anchored. Indeed, more than two-thirds of the genes mapped in this study were located on the same or an overlapping BAC clone as another gene. In a recent analysis of 454 survey sequences, Wicker et al. [16] found evidence of sequence from 3,467 different genes or gene fragments in chromosome 1AL, of which 1352 were syntenic with *B. distachyon,* sorghum and rice. In this study, after taking into account genes that were identified twice with 2 different markers, 1231 gene sequences were placed on the physical map, with a similar ratio of syntenic genes (381/1231). Of the remaining 850 gene sequences, a significant proportion had homologs in the chromosomes of other grasses that are not considered syntenic with 1AL. Some may have been introduced into 1AL by large-scale duplications or translocations from other chromosomes, and so represent smaller scale syntenic blocks. For example, in similarity searches against sorghum and rice about 10% of homologous genes were found on chromosomes Sb03 and Os01 respectively (Figure 4B, C). These chromosomes are syntenic with each other and with the telomeric regions of *B. distachyon* chromosome 2 [19], where 56 homologs of 1AL sequences were also found, suggesting that these regions may contain smaller blocks syntenic with 1AL. However, further mapping would be required to establish the physical location of this putative block on the chromosome. More markers would also be required to locate the other genes identified by the 454 sequences; there is also a possibility that some of these sequences are pseudogenes which have a similar enough sequence to an active gene on chromosome 1B or 1D to have been mis-identified here. Sequencing of the gene encoding regions will be required to resolve this issue. Taking into account the different sizes of the chromosomes, the number of genes identified by transcriptome microarray hybridization (1122) was in a similar range to that found for chromosome 3B (2924 gene loci on a 1GB chromosome) but suggests a slightly lower density of transcribed genes. The genes that have been identified here will be useful in identifying candidate genes for important QTLs on chromosome 1AL, and accelerating marker-assisted breeding programs.

The availability of the BAC-end sequences will be of great value in further refining the physical map; the fact that over half of the 1AL BES contain potential ISBP marker sequences [1] means that it will be possible to design markers specific to the unanchored contigs and thus fill in the gaps in the scaffold for future sequencing of chromosome 1AL. However, as mentioned above the portion of the chromosome that is already anchored is likely to contain the majority of protein-coding genes, and suggests regions of particular interest for pilot sequencing studies.

The conservation of gene order between wheat and the sequenced grass species proved extremely useful in developing a putative order of the contigs in the physical map. Comparison of the syntenic map with deletion bin-mapped and genetic map data (Figure 2) showed a good level of consistency, demonstrating the usefulness of this approach in characterizing large grass genomes. We were able to integrate positional information from several different marker types and bioinformatic analysis to provide the first high-resolution physical map of chromosome 1AL. Based on the physical map, we were able to characterize a significant proportion of the gene space of this chromosome arm (Figure 6). We found evidence that the order of syntenic genes proposed based on the *B. distachyon* gene sequence is remarkably well conserved in 1AL, although sections of the sequence have undergone rearrangement. Also, there are non-syntenic genes interspersed with most of the co-linear genes, and a fraction of the syntenic genes have been translocated to new locations in the chromosome. Overall, the data shows a gradient of increasing recombination along the chromosome arm from the centromere to the telomere, and also that the highest concentration of genes is in the interstitial region of chromosome 1AL (50 cM from the centromere) where there is relatively little recombination.

## Materials and Methods

### BAC library construction, fingerprinting and automated assembly

Plant material, chromosome isolation from *T. aestivum* and construction of two BAC libraries giving a total of 15.7x coverage of chromosome 1AL was previously described [1], [4]. A total of 92,544 BAC clones with an average insert size of 105 kb was fingerprinted using the SNaPshot^TM^ High-Information Content Fingerprinting (HICF) procedure [20]. The number of BAC clones giving good quality fingerprint data was 72,724. These fingerprints were checked for cross-contamination between adjacent wells using GenoProfiler 2.0 software [21] and 2187 suspect fingerprints were eliminated, leaving a total of 70,537 fingerprints (76.2% of the original BAC clones). Initial assembly of the fingerprints was carried out using FPC software [22] with settings adjusted for the complex and repetitive wheat DNA as described previously [3]; briefly, an initial assembly of all clones was carried out under extremely stringent conditions (Sulston Score probability cutoff  = 1e^−75^) to provide a core of high-confidence contigs. The stringency was then relaxed incrementally in steps of −5, adding more clones into the established contigs and merging contigs at each stage, until a cutoff of 1e^−45^. For manual merging, the ends of contigs were compared at a relaxed stringency of 1e^−25^. Contigs were only considered for merging if a) a unique and reciprocal relationship was detected between the ends of the contigs, and b) 2 clones from the end of each contig gave significant matches at this stringency OR a single clone match was supported by marker data. In these cases the CB (Consensus Band) map for the putative merged contig was calculated, and the merge rejected if the resulting map contained >10% Q-clones or other obvious structural defects. Finally, pairs of contigs harboring the same molecular markers (see below) were merged even in the absence of matching clones, giving a contig containing a gap. Unanchored short contigs (consisting of <6 clones and/or less than 200 kb in length) were deemed uninformative, and removed.

### Minimum tiling path and BAC pooling

From the initial automated assembly at a cutoff of 1e^−45^, a Minimum Tiling Path (MTP) consisting of 7470 clones covering the assembly was generated using the MTP module of FPC [17] with the following parameters: minimum FPC overlap 30, maximum overlap 250, FromEnd  = 0, minimum shared bands 12, and preference given to large clones. BAC clones comprising the MTP were picked from the initial library and re-gridded into a 20×384-well plate MTP library as previously described [3]. To facilitate screening of the MTP library a 3D pooling approach was adopted. Each clone was grown independently and then pooled, giving a total of 16 row, 24 column and 20 plate pools, along with a single superpool containing all the clones. After organizing the pooled BAC clones into a single 96-well plate, DNA was amplified from each pool by the multiple displacement method with random primers and phi29 DNA polymerase (GenomiPhi V2 DNA polymerase Kit, GE Healthcare). Pools were diluted 1∶150–1∶200 in PCR Grade water before screening.

### LTC assembly and LTC-check of the FPC assembly

Fingerprinted 70,537 BAC clones were also assembled with LTC software [6]. Briefly, a net of significant clone overlaps were generated with a liberal cutoff of 10^−15^. The average number of overlaps per clone was 15.87, indicating a sufficient stringency cutoff. Prior to clustering, Q-clones and Q-overlaps were removed at cutoffs of 10^−15^ and 10^−25^. Then, first round of adaptive clustering was performed starting from the liberal cutoff of 10^−15^ and, for non-linear clusters, reducing the cutoff by a factor of 1000 each step for a total of 6 steps, to reach a maximum stringency cutoff of 10^−33^. Non-linear clusters that persisted at this stringency were individually visualized to identify problematic clones. A total of 46 clones causing non-linearity were identified and excluded from the second round of adaptive clustering. All other parameters were used as default.

Additionally, the FPC-built assembly was tested with LTC. Final assembly was loaded into LTC software and steps were followed as directed. Testing was performed twice; once without selecting ‘Use maximally connected sub-cluster’, to identify non-connected contigs, and once using maximally connected sub-clusters to identify non-linear contigs.

### Marker selection and development

Markers that had already been genetically mapped to chromosome 1AL were retrieved from the GrainGenes online database (http://wheat.pw.usda.gov/GG2/index.shtml). These included a variety of SSR [23], [24] and EST markers [25]. In addition, Conserved Ortholog Set (COS) markers were developed for the group 1 chromosomes from wheat EST sequences as previously described [7]. To populate the non-gene coding sections of the chromosome with genetic markers, BAC-end sequencing had already been carried out for the MTP clones and putative insertion-site based polymorphisms (ISBP) identified in the BESs [1]. In addition to the markers reported in that paper, a further 280 ISBPs were selected based on the following criteria designed to avoid marker redundancy: 1) a high-confidence junction between repetitive and non-repetitive DNA; 2) junctions where the end of the repetitive element could be clearly identified; 3) if a particular repetitive element occurred more than once in the BESs, only one possible ISBP was selected from all occurrences.

### PCR screening and analysis

The 3D pools were screened for the molecular markers designed above using *Taq* polymerase (Fermentas) or *i-*Taq (Intron) and standard PCR protocols. From each pool 1-2 µl of template DNA was used in a 10 µl reaction volume. PCR products were analyzed by agarose gel electrophoresis and the positive pools used to reconstruct the original MTP clone ID. When a given marker was found in more than one row, column and plate pool, further PCRs were carried out using the indicated MTP clones as template (sampled directly from deep frozen BAC library plates). Positives were then compared with a correspondence list to find the address of the clone(s) in the original BAC library.

### Hybridization to a wheat transcriptome microarray

The wheat NimbleGen 40 K transcriptome microarray was designed based on the NCBI *T. aestivum* Unigene set build #55 and contained an average of 3 different 60-mer sequence probes for each of 39179 Unigenes [15]. Labelling of the MTP pool DNAs was carried out using the NimbleGen Dual Colour labelling kit (Roche NimbleGen, Inc.) according to the manufacturer's recommendations. Each of the 60 pools was labelled separately with Cy3 and Cy5 and then pairs of pools were hybridized together in a dye-swap design. Hybridization for 72 hours at 42°C and washing and drying of the arrays were carried out according to the manufacturer's guidelines, and arrays were scanned immediately after drying using an MS 200 microarray scanner (Roche NimbleGen, Inc.) at 2nm resolution with automatic gain adjustment. Scanned images were aligned and analyzed with DEVA software. Signal intensities were extracted from scan data and normalized and deconvoluted using automated R scripts (www.r-project.org), as described previously [15]. Briefly, within each pool, if one probe among the 3 for each Unigene was found to be an outlier it was removed from the dataset. Pools were then normalized with respect to each other by subtracting the median and dividing by the standard deviation of all signal intensities within the pool.

### Statistical analysis of microarray data

Three files were generated containing the normalized signal intensities for row, column and plate pools respectively, and two complementary statistical methods used to identify positives within each pool type. In the first method, a signal was scored as positive if the median intensity for all the probes for a single gene in a single pool exceeded [Mean + C × Standard Deviation] of the intensities for that gene across all the pools, where C was a pre-defined threshold co-efficient. Three different thresholds were calculated for each pool type: High (C  = 2.6 for row pools, 2.8 for column and plate pools), Medium (C  = 2.4 for row pools, 2.6 for columns and plates) and Low (C = 2.2 for row pools, 2.4 for columns and plates). The second method used Student's t-Test to determine whether the intensities for a given gene in one pool were significantly different from all the other pools, assuming variances to be equal. Again High, Medium and Low thresholds were defined (p<0.01, p<0.025 & p<0.05 respectively). Unigenes were assigned to specific BACs if they passed both tests at the High threshold for a unique row, column and plate pool (869 in total). Where more than one of any of the three pool types passed the high threshold for both tests, the Unigene was allocated to BACs only if the physical map contained 2 overlapping BACs that would give positive signals in the observed pools. Similarly, Unigenes found at the medium threshold were added only if they were present on 2 physically overlapping BACs and had not been identified at the high thresholds; finally Unigenes found at the low thresholds were added only if they were found on 2 physically overlapping BACs and had not been identified at the high or medium thresholds. By this means a total of 253 Unigenes were assigned to overlapping pairs of BACs, bringing the total number of Unigenes located on the physical map to 1122.

### Similarity searches, annotation and synteny

All similarity searches were carried out using the BLAST+ stand-alone toolkit, version 2.2.24, from the NCBI [26]. Functional annotation of Unigene sequences was carried out using Blast2GO software [27] as follows: the Unigene sequences were searched against the non-redundant protein database at http://www.ncbi.nlm.nih.gov/Blast.cgi using blastx, with a maximum e-value cutoff of 1e^−10^ and a minimum high-scoring pair length cutoff of 33. The best 20 hits for each sequence were retained and used for GO-mapping. After GO terms had been mapped to each sequence, automated annotation was carried out using an annotation score cutoff of 50 and an HSP-Hit coverage cutoff of 30% and other parameters at default values. The annotation was streamlined using GO-Slim_Plant to limit the results to plant-relevant nodes.

For detection of synteny with sequenced grass genomes, coding sequence datasets were downloaded from PlantsDB (http://mips.helmholtz-muenchen.de/plant/genomes.jsp) for the *Brachypodium distachyon* genome annotation v1.2 [19], *Sorghum bicolor* genome annotation v1.4 [28], and version 5 of the *Oryza sativa* pseudomolecules [29; rice.plantbiology.msu.edu/]. Similarity searches were carried out using blastn for higher stringency, with an empirically determined e-value cutoff of 1e^−25^, and any hits shorter than 50 nt were rejected. The highest scoring hit for each UniGene sequence was retained. The syntenic gene matches were ordered using the virtual gene order for the group 1 chromosomes produced by Mayer et al. [5].

## Supporting Information

Figure S1 **Network diagrams of non-linear contigs in assembly of 1AL BAC clones by LTC (DOC).**
(DOC)

Figure S2 **Examples of non-linear FPC contigs identified by LTC, and recommended solutions (DOC).**
(DOC)

Table S1 **Primer sequence and mapping details of all new COS and ISBP markers designed and tested during the course of this study (XLS).**
(XLS)

Table S2 **Details of all UniGenes mapped to 1AL BAC clones (XLS).**
(XLS)

Data S1 **Summary of features of the FPC contig assembly that were not consistent with LTC (XLS).**
(XLS)
