# Supplementary material for: Physical Mapping Integrated with Syntenic Analysis to Characterize the Gene Space of the Long Arm of Wheat Chromosome 1A
Source: PLoS One. 2013 Apr 16;8(4):e59542. doi: 10.1371/journal.pone.0059542 (PMC3628912; doi:10.1371/journal.pone.0059542)
Supplement: Figure S1 — Network diagrams of non-linear contigs in assembly of 1AL BAC clones by LTC (DOC). (DOC) [file pone.0059542.s001.doc]

**
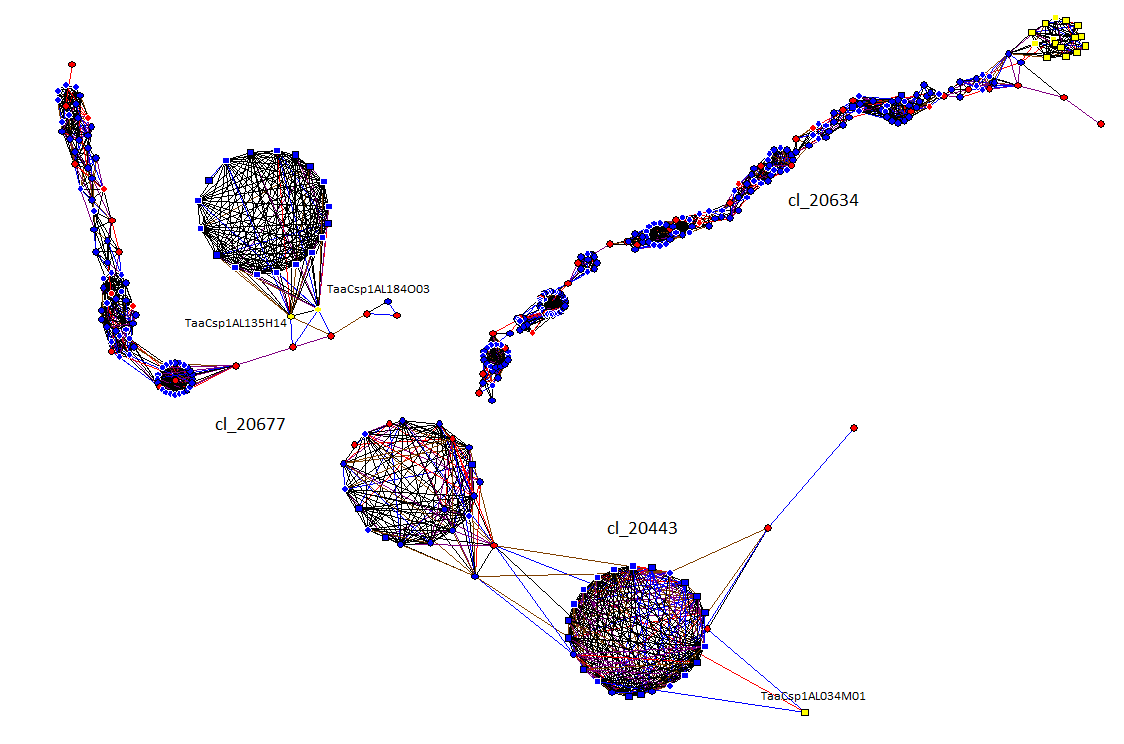
**

**Figure S1: Non-linear contigs in assembly of 1AL BAC clones by LTC**

Contig structures are represented as a net of significant clone overlaps, with each vertex representing a clone and each line a significant overlap between 2 clones. Red circles represent MTP clones, while squares denote clones that have no direct overlap with the MTP. Shapes with a white border denote buried clones. For contigs cl_20677 and 20634, the non-linearity is likely to be the result of the MTP selection by LTC, which can be fixed via the manual selection of an alternative MTP. For cl_20634, a few clones from a highly-connected group of clones, highlighted in yellow, could be added to the MTP. In cl_20677, however, since TaaCsp1AL184O03 is a buried clone, TaaCsp1AL135H14 is effectively the only clone linking the circular group of interconnected clones to the rest of the contig. Removal of these two clones (highlighted in yellow) could be considered, which would split the contig into two linear sub-contigs. For cl_20443, the non-linearity stems from a single clone (TaaCsp1AL034M01 – highlighted in yellow) in which case this clone is likely to have a low-quality fingerprint, and no further action is required.
