# Supplementary material for: Physical Mapping Integrated with Syntenic Analysis to Characterize the Gene Space of the Long Arm of Wheat Chromosome 1A
Source: PLoS One. 2013 Apr 16;8(4):e59542. doi: 10.1371/journal.pone.0059542 (PMC3628912; doi:10.1371/journal.pone.0059542)
Supplement: Figure S2 — Examples of non-linear FPC contigs identified by LTC, and recommended solutions (DOC). (DOC) [file pone.0059542.s002.doc]

**Figure S5. Examples of non-li near FPC contigs discovered by LTC, and recommended solutions.** The network of significant clone overlaps for each contig as generated by LTC is shown. Each vertex represents a clone and each line a significant overlap between 2 clones; red circles represent MTP clones, while squares denote clones that have no direct overlap with the MTP. Shapes with a white border denote buried clones.


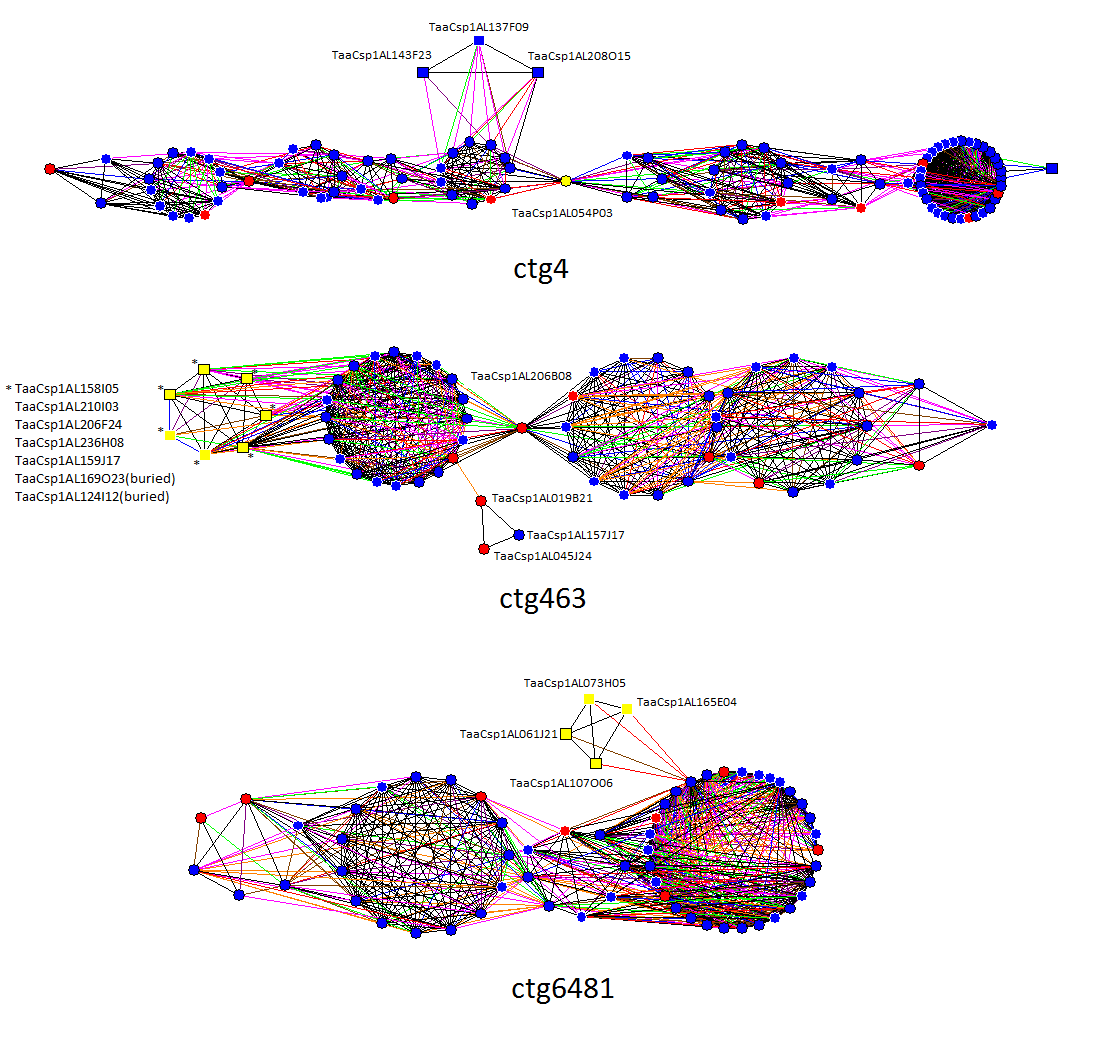


In **ctg4**, clones TaaCsp1AL208O15, TaaCsp1AL137F09 and TaaCsp1AL143F23 are suggested to disrupt the linearity of the contig by the LTC program. Low-quality fingerprinting of these clones may have resulted in the disconnection of these clones from the MTP. However, it is also possible that clone TaaCsp1AL054P03 (highlighted in yellow) is chimeric, putatively linking two different parts of the chromosome arm. Further work on this contig may require omission of TaaCsp1AL054P03 clone and splitting the contig into two linear parts.

For **ctg463**, the MTP selection (red vertices) appears to exclude 7 clones from the MTP; these clones are highlighted in yellow and listed to the left. An alternative MTP selection including the non-buried clones of these seven would result in a linearized net of significant clone overlaps, with clones TaaCsp1AL019B21, TaaCsp1AL045J24 and TaaCsp1AL157J17 constituting a separate cluster as the connection of TaaCsp1AL019B21 with the main cluster appears weak. In addition, TaaCsp1AL206B08 may be a chimeric clone; however it also might be correct, and just come from a low-coverage area of the chromosome arm.

For **ctg6481**, four clones, namely, TaaCsp1AL165E04, TaaCsp1AL073H05, TaaCsp1AL061J21 and

TaaCsp1AL107O06 appear to deviate from the linearity of the contig. When this contig was re-calculated following changes to other parts of the assembly, TaaCsp1AL107O06 was included in the MTP, removing any non-linearity. However, as these 4 clones only overlap significantly with a single clone in the rest of the contig, additional sequence-level analysis should be carried out to confirm whether or not they are really part of this contig.
